# Maintenance of elderly AML patients with androgens

**DOI:** 10.18632/oncotarget.21447

**Published:** 2017-10-02

**Authors:** Arnaud Pigneux, Marie C. Béné

**Affiliations:** Arnaud Pigneux: Hematology Clinic, Bordeaux University Hospital Haut Levêque, Pessac, France

**Keywords:** AML, elderly, androgens, survival and improving

Acute myeloblastic leukemia (AML), although liable to occur at any age (including children) is however mostly a disease of the elderly. The ongoing progression of human life-span makes it likely that this pathology will be of growing importance in the coming years. It is therefore a clear public health issue to find ways of efficiently treating these patients while preserving as much as possible their quality of life. Of note, although treatment standards have been nearly the same for more than 50 years, the life expectancy of AML in elderly patients has nonetheless been steadily improving. This can be attributed to a better management of treatment-associated toxicities, but also to the globally improved health of the ageing population. Several issues have been considered to improve the complete remission (CR) rate of these patients and prolong survival. For the first of these goals, the GOELAMS group (now called FILO), following a study performed in the south of France in the nineties [1], has advocated the addition of Lomustine in the induction schedule. A larger scale trial in which lomustine was also added to consolidation was next performed to this avail, currently being submitted for publication [Pigneux et al. ASH 2015, # 3736]. In a recent publication from our group [2] a lomustine-based induction was followed, as soon as patients were recovering from aplasia, by the randomized addition, or not, of the androgen norethandrolone, prolonged, as maintenance therapy, for two years after CR achievement. Two important points must be highlighted from this study. First, the excellent CR rate obtained after induction, likely related to the addition of lomustine to a standard schedule [3]. Second, the positive effect of norethandrolone maintenance only apparent and statistically significant one year after diagnosis. This puzzling observation, which imposed the use of time-dependent statistical analyses, however proved to be statistically significant, with even the hint of a plateau for patients in the norethandrolone arm. This delay in survival improvement can be interpreted as the time needed for normal hematopoiesis to recover after a rather solid chemotherapy. Indeed, early deaths, in this ageing population, was at an expected rate (16% of early deaths during induction and 8% of induction failure) mostly occurring in patients with unfavorable cytogenetics). However, for patients who successfully came out of this tough initial period, the benefit of norethandrolone addition to maintenance therapy became very clear. Indeed, five year PFS was 31.2% versus 16.2 for patients who did not receive norethandrolone (*p* = 0.02).

Although the trial, initiated in 2002, did not include any advanced biological exploration of the effect of norethandrolone, this difference calls for attention. One of the plausible explanations would be that norethandrolone mostly acted on residual normal hematopoietic stem cells, inducing them to improve hematopoietic recovery. This is consistent with the benefit of androgens in the treatment of aplastic anemia [4] where they are likely to trigger healthy hematopoiesis from dormant stem cells. Several studies have suggested that this could be related to the specific trigger of telomerase by androgens [5]. However, as mentioned in the trial’s publication [2], transcripts of the androgen receptor have been found in the MILE study [6] to be at higher levels in AML patients than in controls, suggesting a sensitivity of blast cells to the addition of androgens. Unfortunately, minimal residual disease investigation was not available in the LAMSA2002 trial, which would possibly have confirmed an increased depletion in malignant cells by the addition of norethandrolone, based on this observation of higher transcripts of the receptor.

All in all, and even if further studies are obviously needed to better understand the whereabouts of this observed and clear beneficial effect, it appears that the addition of androgens could be an interesting option in the management of elderly AML patients, and perhaps also younger ones. One remaining issue is the potential specificity of norethandrolone in achieving this improved outcome, since this drug is not universally available.
